# Repository corticotropin injection in patients with persistently active SLE requiring corticosteroids: post hoc analysis of results from a two-part, 52-week pilot study

**DOI:** 10.1136/lupus-2017-000240

**Published:** 2017-12-27

**Authors:** Richard A Furie, Margaret Mitrane, Enxu Zhao, Patrice M Becker

**Affiliations:** 1 Hofstra Northwell School of Medicine, Northwell Health, Great Neck, New York, USA; 2 Manhattan BioPharm Consultants LLC, New York, New York, USA; 3 Mallinckrodt ARD Inc., Bedminster, New Jersey, USA

**Keywords:** clinical research, corticotropin, systemic lupus erythematosus, disease activity, Clinical Trials

## Abstract

**Objective:**

Post hoc analyses evaluated the effectiveness and safety of repository corticotropin injection (RCI) in patients with persistently active SLE over 52 weeks.

**Methods:**

Patients were initially randomised to 40 U daily or 80 U every other day RCI (n=26) or placebo (n=12) for the 8-week double-blind period. Completers entered the open-label extension (OLE; n=33) receiving 16, 40 or 80 U RCI 1–3 times/week and were followed through week 52. Outcomes included proportion of responders based on a novel index (resolution of joint or skin activity using hybrid Systemic Lupus Erythematosus Disease Activity Index (hSLEDAI) without any worsening British Isles Lupus Assessment Group (BILAG) scores in other organ systems) or revised novel index (using SLE Responder Index (SRI) definition of BILAG worsening (1A or 2B)), proportion of responders by SRI and changes in total hSLEDAI and BILAG scores. Adverse events and laboratory values were assessed.

**Results:**

At week 52, 12.0% (3/25) RCI/RCI patients and 36.4% (4/11) placebo/RCI patients were responders using the novel index. The revised novel responder index demonstrated response rates of 48.0% (12/25) and 54.5% (6/11) in the RCI/RCI and placebo/RCI groups, respectively. Proportions of SRI responders were 40.0% (10/25) and 54.5% (6/11). In the RCI/RCI group, total hSLEDAI and BILAG scores declined from 10.0 and 15.7 at week 0 to 3.5 and 4.6 at week 52, respectively. Reductions in the placebo/RCI group on switching were observed (mean hSLEDAI: 9.1–3.3; BILAG: 13.5–2.6). Other disease activity endpoints also improved in both groups. No new safety signals were observed during the OLE.

**Conclusions:**

RCI demonstrated durable effectiveness in patients with persistently active SLE despite moderate-dose corticosteroid therapy. Switching from placebo resulted in reduced disease activity during the OLE. These data provide the foundation for evaluation of RCI in a robustly powered study.

**Key messages**

- This post hoc analysis of results from a two-part pilot study consisting of an 8-week, prospective, randomised, double-blind, placebo-controlled phase, followed by a 44-week open-label extension (OLE), demonstrated sustained improvements in signs and symptoms of SLE in patients treated with repository corticotropin injection (RCI; H.P. Acthar Gel) for a total of 52 weeks.
- Patients who switched from placebo to RCI at the beginning of the OLE experienced improvements in signs and symptoms, which were comparable with those for patients who had received RCI in the first 8 weeks of the study.
- No new or unexpected safety or tolerability signals were observed during the OLE when compared with the double-blind period.

## Introduction

SLE is a systemic autoimmune disease that results in significant and irreversible damage to multiple organs.^1^ It is characterised by the deposition of immune complexes, and inflammation and organ damage are exacerbated by infiltration of leucocytes and the release of proinflammatory chemokines and cytokines.^1–3^ The aetiology of lupus is multifactorial, but B lymphocyte hyperactivity and loss of tolerance are major drivers of the disease.^1^ Affecting about 14–68 in every 100 000 persons in the USA,^2^ with similar prevalences in other parts of the world,^4 5^ SLE is associated with significant morbidity and mortality.^6^

Patients with SLE may be treated with non-steroidal anti-inflammatory drugs, corticosteroids, hydroxychloroquine, methotrexate, azathioprine, ciclosporin, mycophenolate mofetil, tacrolimus, leflunomide or B lymphocyte-directed monoclonal antibodies (eg, rituximab and belimumab).^5 7^ Despite these options, there are important unmet treatment needs in SLE: conventional immunosuppressive agents have significant toxicities, and biological agents in randomised controlled trials have had modest effects, at best.^8^

Adrenocorticotropic hormone (ACTH) has been used since the 1950s for the treatment of patients with SLE.^9^ ACTH stimulates cortisol production by the adrenal gland^10^ and also targets melanocortin receptors that are present on immune cells.^10 11^ Additionally, ACTH may increase levels of the anti-inflammatory cytokine interleukin (IL)-10 and decrease B lymphocyte proliferation and differentiation, as reflected by significant reductions in splenic B lymphocyte follicular and germinal centre cells and decreased levels of anti-double-stranded DNA (dsDNA) autoantibodies in a rodent model of lupus.^11 12^ Additional data suggest that RCI, but not placebo, attenuated IL-4/CD40 ligand-induced proliferation and immunoglobulin production in B lymphocytes isolated from healthy human volunteers.^13^

Repository corticotropin injection (RCI; H.P. Acthar Gel, Mallinckrodt ARD Inc., Bedminster, New Jersey, USA) is a prolonged-release formulation containing a highly purified porcine ACTH analogue. The US Food and Drug Administration has approved RCI for a number of indications, including for use during SLE exacerbations or as maintenance therapy in selected patients with SLE.^14^ Results were recently reported for the double-blind and open-label extension (OLE) of a pilot study in patients with persistently active SLE, including rash and/or arthritis, despite treatment with moderate-dose corticosteroids (NCT01753401).^15 16^ Although the primary endpoint of the double-blind portion of the study was not met, there were improvements in several other measures of disease activity for RCI versus placebo, including total hybrid Systemic Lupus Erythematosus Disease Activity Index (hSLEDAI), total British Isles Lupus Assessment Group (BILAG), Cutaneous Lupus Erythematosus Disease Area and Severity Index (CLASI) activity scores and tender and swollen joint count.^16^ This report summarises post hoc analyses that evaluated the efficacy of RCI over the entire 52-week study.

## Methods

### Study design

**Figure 1:**
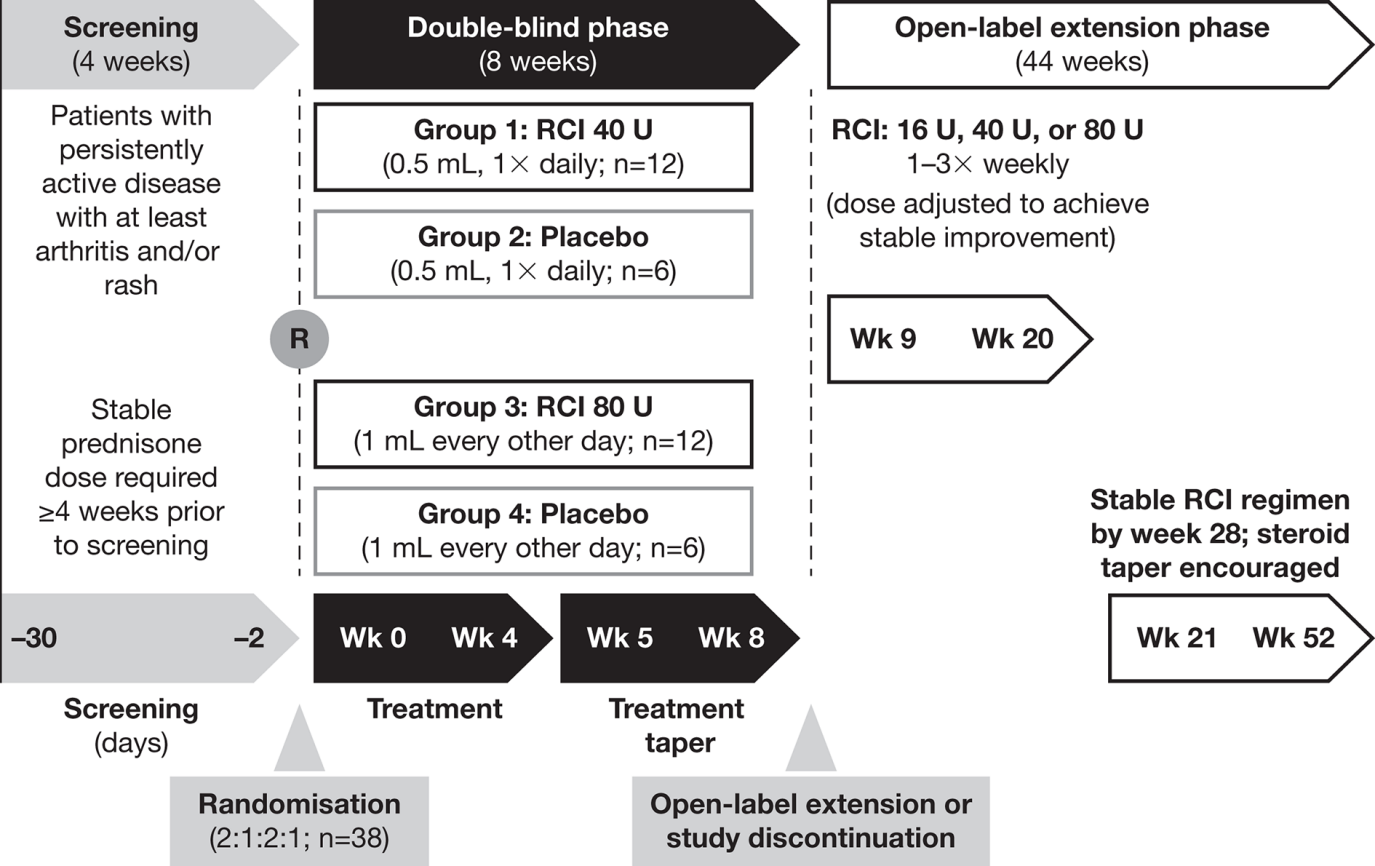
Study design. R, randomisation; RCI, repository corticotropin injection; Wk, week.

This phase 4 pilot study (NCT01753401) consisted of an 8-week, randomised, double-blind, placebo-controlled treatment period followed by a 44-week OLE (figure 1).^16^ The first period explored the effect of RCI on disease activity in patients with SLE and persistently active arthritis and/or rash despite standard of care that included chronic/stable prednisone use for a minimum of 4 weeks before screening. The study was conducted at 20 sites in the USA. All patients provided written informed consent. All principal investigators, subinvestigators and study coordinators at each site were required to complete scale assessment training prior to enrolling any patients. Because this was a pilot study, serial assessments done by a single examiner were not required.

### Patients

Inclusion and exclusion criteria for the double-blind period have been described.^16^ Briefly, adult patients (age ≥18 years) were eligible if they met at least four of the American College of Rheumatology-revised diagnostic criteria for SLE^17^ and had active disease with joint and/or skin involvement sufficient to score ≥2 on the hSLEDAI. Patients were also required to have a BILAG^18^ score of A or B in the mucocutaneous and/or musculoskeletal domains. All patients were required to be seropositive for, or have a documented history of, antinuclear antibodies and any one of the following: anti-dsDNA, anti-Smith or anticardiolipin antibodies. Persistent disease activity had to be evident despite the use of stable, moderate-dose corticosteroids (prednisone 7.5–30 mg/day or equivalent) for at least 4 weeks prior to screening. All patients completing the double-blind period were eligible to enter the OLE phase.

### Interventions

In the double-blind phase, patients were assigned randomly to receive RCI 40 U daily or 80 U every other day by subcutaneous injection or to one of two volume-matched placebo groups. During weeks 5–8 of the double-blind phase, RCI doses were tapered by reducing the frequency of administration to twice weekly according to a protocol-defined schedule. The initial RCI dose in the OLE was based on the study drug regimen at the completion of the double-blind period at week 8. Patients receiving 0.2 mL (RCI 16 U), 0.5 mL (RCI 40 U) or 1.0 mL (RCI 80 U) injections of RCI or placebo at week 8 received RCI 16 U, 40 U or 80 U twice weekly at the start of the OLE. Investigators were permitted to adjust the RCI regimen for safety or efficacy reasons from weeks 9–20 to achieve a stable RCI regimen (defined as consistent volume and frequency over at least 8 weeks) no later than week 28. Once a stable RCI regimen was achieved, steroid taper was encouraged but left to the investigator’s judgement. The goal was to taper completely off steroids, but if this was not possible, attempts were made to taper to a low (<7.5 mg/day) daily dose.

### Assessments

Efficacy variables included the hSLEDAI,^19^ BILAG-2004, Physician’s Global Assessment (PGA),^20^ Safety of Estrogens in Lupus Erythematosus National Assessment (SELENA) Flare Index (SFI),^20–22^ CLASI activity score^23^ and tender and swollen joint count. The hSLEDAI is identical to the SELENA-SLEDAI but uses the SLEDAI-2K definition for proteinuria and was used because it is able to capture ongoing proteinuria. Efficacy assessments were performed every 4 weeks except for hSLEDAI and SFI, which were assessed every 2 weeks for the first 8 weeks, then every 4 weeks for the remainder of the study. Safety was assessed by monitoring adverse events (AEs), physical examinations, measurements of vital signs and clinical laboratory investigations.

### Outcome measures

A novel composite responder index, which was evaluated at 4-week intervals, defined a responder as a patient with a decrease in hSLEDAI score from 4 to 0 for arthritis and no worsening in other organ systems based on BILAG score, or a patient with a decrease in hSLEDAI score from 2 to 0 for rash and no worsening in other organ systems based on BILAG score. Worsening was defined as an increase in any BILAG score to a higher level (eg, a minor change in musculoskeletal or mucocutaneous systems could increase a D score to C, which was considered worsening). Because of the stringency of the definition of ‘worsening’, a revision to the novel composite endpoint was made. The revised novel composite responder index redefined a responder as a patient with a decrease in hSLEDAI score from 4 to 0 for arthritis or from 2 to 0 for rash and no new BILAG A score and no more than one new BILAG B organ domain score. Additional assessments included the proportions of patients meeting the SRI, hSLEDAI decrease ≥4 and rate of severe SFI flares at weeks 8 and 52. Other disease activity outcomes included changes in hSLEDAI, total BILAG score, PGA, changes in tender and swollen joint count for patients with score >0 at double-blind baseline and CLASI activity score for patients with score >0 at double-blind baseline. The proportion of patients achieving a reduction in daily prednisone dose to <7.5 mg/day by week 52 was also determined. Safety endpoints included AEs, serious AEs (SAEs) and SAEs and AEs leading to treatment discontinuation over the 52-week period.

### Statistical analyses

All results reported used the modified intent-to-treat (mITT) population, defined as all randomised patients who had received at least one dose of study medication and who had any post-baseline efficacy or safety data. Descriptive statistics were used for continuous and categorical data. Quantitative endpoints were summarised using means and SD at each time point, and categorical endpoints were summarised using frequency counts and percentages at each time point. Non-responder imputation was used for missing data in the responder analyses. Analyses were performed using SAS V.9.3 software (Cary, North Carolina, USA).

## Results

### Patients

**Figure 2:**
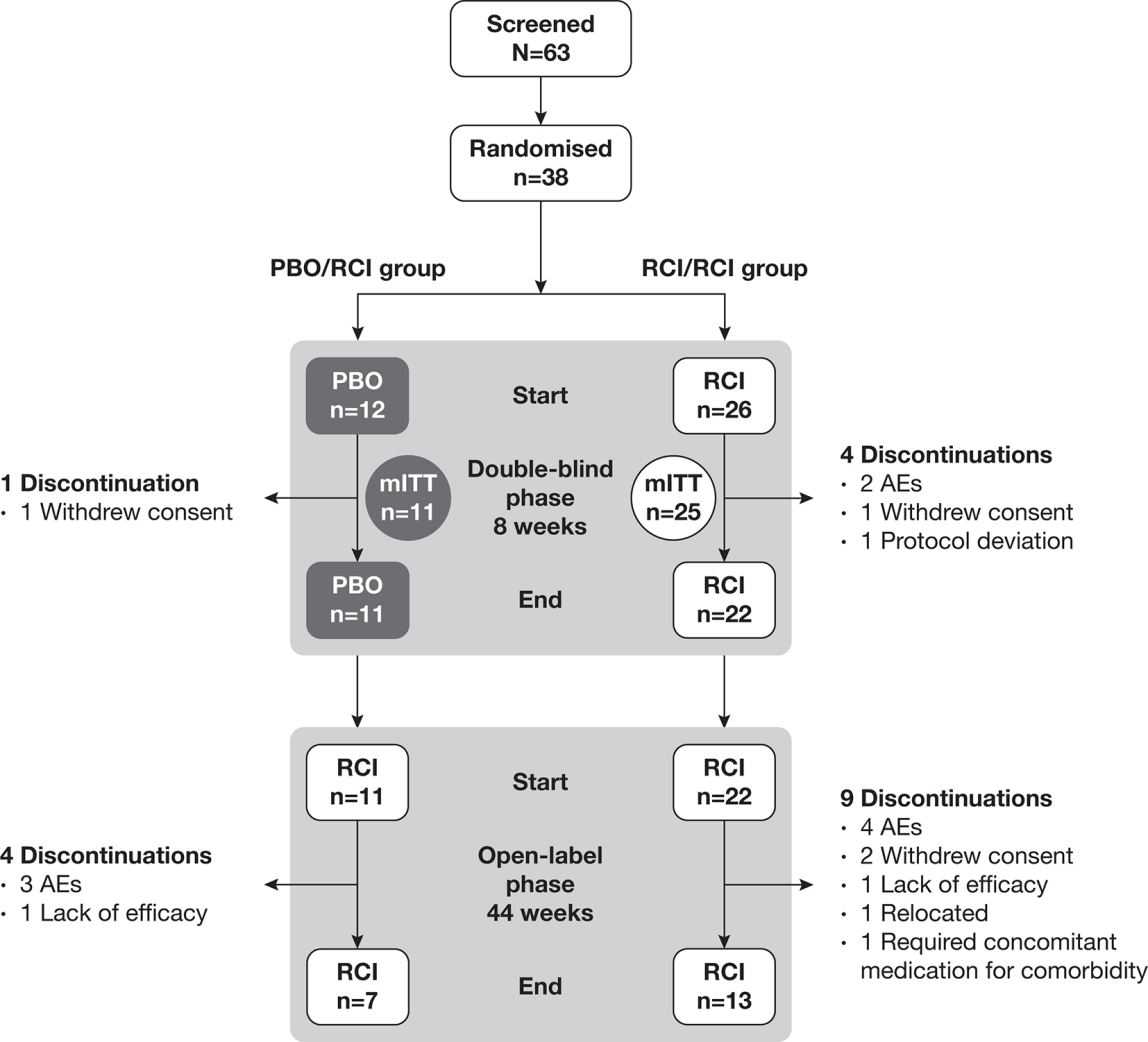
Patient disposition. AE, adverse event; mITT, modified intent-to-treat; PBO, placebo; RCI, repository corticotropin injection.

A total of 38 patients were randomised to RCI (n=26) or placebo (n=12) for the double-blind period; 33 completed visits through week 8 and entered the OLE (RCI/RCI: n=22, placebo/RCI: n=11), and 20 completed visits through week 52 (RCI/RCI: n=13, placebo/RCI: n=7; figure 2). During the 52-week treatment period, 13 patients in the RCI/RCI group and 5 patients in the placebo/RCI discontinued treatment. Two patients (one in each group) withdrew consent and were not included in the mITT population. The mITT population consisted of 36 patients.

Demographic and disease characteristics at double-blind baseline have been presented previously.^16^ Twelve patients (33.3%) reported the use of immunosuppressant medications (azathioprine, methotrexate or mycophenolate mofetil) during the 52-week study period and 26 (72.2%) used antimalarials. The mean prednisone daily dose was 10.0 mg/day in the RCI/RCI group and 16.4 mg/day in the placebo/RCI group. All measures of disease activity were higher for the placebo/RCI group versus the RCI/RCI group at the OLE baseline (table 1).

**Table 1:** Efficacy endpoints at weeks 8 and 52 (mITT population)

	Week 0 (baseline)		Week 8 (OLE baseline)		Week 52	
	RCI/RCI	Placebo/RCI	RCI/RCI	Placebo/RCI	RCI/RCI	Placebo/RCI
Total hSLEDAI	10.0 (3.3)	9.8 (2.1)	5.8 (3.0)	9.1 (3.4)	3.5 (3.5)	3.3 (2.5)
Total BILAG-2004	15.7 (5.9)	15.4 (9.6)	6.8 (4.3)	13.5 (8.8)	4.6 (6.0)	2.6 (2.9)
CLASI activity score*	6.7 (6.3)	7.4 (6.6)	3.9 (4.3)	7.0 (7.0)	1.3 (1.6)	0.5 (0.8)
Tender and swollen joint count*	7.4 (5.8)	5.1 (4.9)	1.4 (2.3)	2.2 (3.2)	0.9 (2.5)	1.6 (2.6)
Physician’s Global Assessment	54.4 (13.0)	52.6 (12.5)	28.7 (21.1)	39.1 (27.2)	15.6 (16.5)	11.7 (13.2)
Novel responder index, n/N (%)	n/a	n/a	11/25 (44.0)	3/11 (27.3)	3/25 (12.0)	4/11 (36.4)
Revised novel responder index,† n/N (%)	n/a	n/a	15/25 (60.0)	4/11 (36.4)	12/25 (48.0)	6/11 (54.5)
SLE responder index, n/N (%)	n/a	n/a	13/25 (52.0)	1/11 (9.1)	10/25 (40.0)	6/11 (54.5)
hSLEDAI decrease ≥4, n/N (%)	n/a	n/a	15/25 (60.0)	1/11 (9.1)	10/25 (40.0)	6/11 (54.5)
Prednisone <7.5 mg/day, n/N (%)	n/a	n/a	n/a	n/a	9/25 (36.0)	3/11 (27.3)
Severe flare (SFI), n/N (%)	n/a	n/a	2/25 (8.0)	1/11 (9.1)	4/25 (16.0)	3/11 (27.3)

Data reported as mean (SD) unless indicated otherwise.

*Calculated for patients with score >0 at double-blind baseline.

†Novel responder index calculated using SRI definition for BILAG worsening (no new BILAG A and not more than one new BILAG B).

BILAG, British Isles Lupus Assessment Group; CLASI, Cutaneous Lupus Erythematosus Disease Area and Severity Index; hSLEDAI, hybrid Systemic Lupus Erythematosus Disease Activity Index; mITT, modified intent-to-treat; n/a, not applicable; OLE, open-label extension; RCI, repository corticotropin injection; SFI, Safety of Estrogens in Lupus Erythematosus National Assessment (SELENA) Flare Index.

### Efficacy

**Figure 3:**
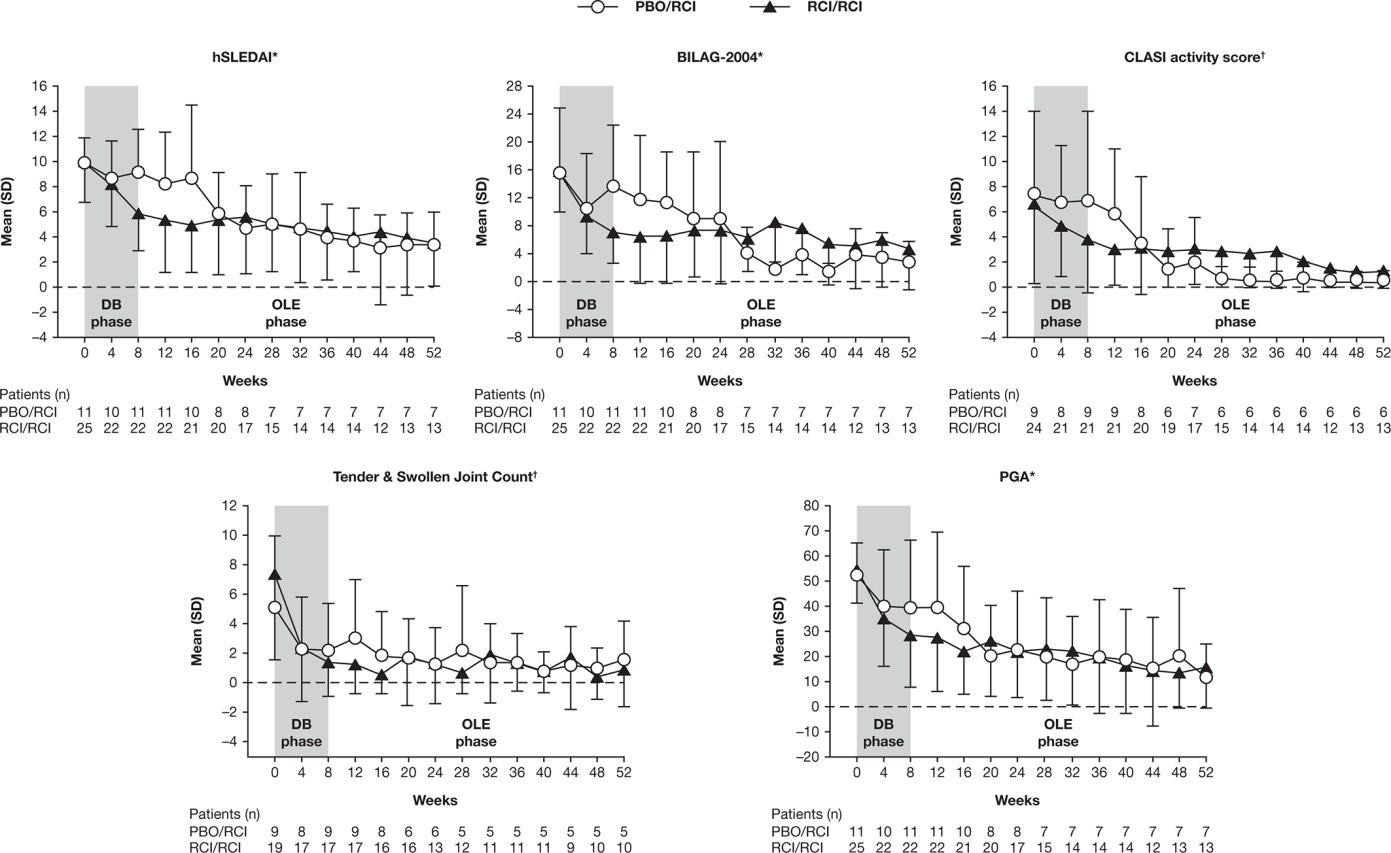
Disease activity measures over time. *Mean and SD were calculated on the basis of the observed data at each time point. †Mean and SD were calculated using only scores >0 at week 0. BILAG, British Isles Lupus Assessment Group; CLASI, Cutaneous Lupus Erythematosus Disease Area and Severity Index; DB, double-blind; hSLEDAI, hybrid Systemic Lupus Erythematosus Disease Activity Index; OLE, open-label extension; PBO, placebo; PGA, Physician’s Global Assessment; RCI, repository corticotropin injection.

The proportions of patients defined as responders in the RCI/RCI group using the novel index at weeks 8 and 52 were 44.0% (11/25) and 12.0% (3/25), respectively. Applying the revised novel composite index (which reduced the unfavourable impact of the stringent BILAG worsening criteria by introducing the SRI definition for worsening in BILAG-2004), the proportions of responders were 60% (15/25) and 48.0% (12/25) at weeks 8 and 52, respectively (table 1). Improvements were also seen with standard disease activity indices (hSLEDAI decrease ≥4 and SRI) at weeks 8 and 52 for the RCI/RCI group. Total hSLEDAI, BILAG-2004, CLASI activity and PGA scores and, to a lesser extent, tender and swollen joint count, continued to improve from weeks 8–52 in these patients (figure 3). The proportion of patients that decreased their daily prednisone dose to <7.5 mg was 36.0% (9/25) at week 52 for the RCI/RCI group (table 1).

For patients who had received placebo during the double-blind period and who switched to RCI for the OLE, disease activity measures improved to levels comparable with those seen in patients in the RCI/RCI group (table 1). The effects of RCI were generally observed on or before 12–16 weeks after the switch from placebo to RCI (figure 3). The proportions of responders at weeks 8 and 52 were 27.3% (3/11) and 36.4% (4/11), respectively, using the novel index and 36.4% (4/11) and 54.5% (6/11) using the revised responder index, respectively (table 1). The proportion of patients that decreased their daily prednisone dose to <7.5 mg was 27.3% (3/11) at week 52 for the placebo/RCI group (table 1).

Of the 12 patients receiving immunosuppressive therapies at the start of the trial, two discontinued concomitant immunosuppressants (mycophenolate mofetil, azathioprine; one patient each) and one reduced their dose of methotrexate from 12.5 mg daily to 7.5 mg during the OLE period.

### Safety and tolerability

The incidences of treatment-emergent AEs (TEAEs) and treatment-related AEs in the placebo/RCI and RCI/RCI groups were similar over the 52-week study period (table 2). One death attributed to *Klebsiella* sepsis, and multiorgan failure was reported in the RCI/RCI group. This event was previously reported.^16^ Three other RCI/RCI patients experienced serious TEAEs (one patient with gastro-oesophageal reflux and chest discomfort (double-blind phase), one with haemorrhagic ovarian cyst and viral infection (double-blind phase) and pelvic infection and lower abdominal pain (OLE) and one with pelvic abscess (OLE)). Of the four patients experiencing serious TEAEs, two were classified as probably or definitely related to the study drug (chest discomfort/gastro-oesophageal reflux and pelvic abscess). Four placebo/RCI patients experienced serious TEAEs (viral infection, non-cardiac chest pain, pyelonephritis and SLE flare with hospitalisation). All of these events occurred during the OLE, and only one (pyelonephritis) was classified as possibly related to study drug.

**Table 2:** Summary of TEAEs and treatment-related AEs over 52 weeks*

Parameter	Placebo/RCI (n=11)	RCI/RCI (n=25)
Any TEAE	11 (100)	23 (92.0)
Any severe TEAE†	3 (27.3)	2 (8.0)
Any treatment-related AE‡	6 (54.5)	14 (56.0)
Any TEAE leading to study discontinuation	3 (27.3)	6 (24.0)
Any serious TEAE	4 (36.4)	4 (16.0)
Any TEAE resulting in death	0	1 (4.0)

Data reported as n (%).

*AEs classified into System Organ Class and preferred terms using the Medical Dictionary for Regulatory Activities, V.15.1.

†AEs were considered severe if the severity of an event was missing.

‡AEs were considered treatment related if the relationship to study medication was possibly related, probably related, definitely related or missing.

AE, adverse event; RCI, repository corticotropin injection; TEAE, treatment-emergent adverse event.

TEAEs leading to study discontinuation occurred in six patients in the RCI/RCI group (three during the double-blind phase and three during the OLE) and included one severe event, a false-positive hepatitis C screening test result (after withdrawal from the study, this patient died of *Klebsiella* sepsis). Of the three TEAEs leading to discontinuation in the placebo/RCI group, one occurred during the double-blind phase and two during the OLE. Only one (ulcerative keratitis during the OLE) was classified as severe and was not related to study drug. There were no clinically significant changes in physical examination findings or vital signs, including blood pressure or clinical laboratory tests, during the 52-week treatment period.

## Discussion

The results of the post hoc analyses for this two-part, 52-week, phase 4 study demonstrated that the addition of RCI to standard of care led to relatively rapid and sustained improvements in a patient population with SLE and refractory arthritis and/or rash despite moderate-dose corticosteroid therapy.

Patients who received RCI throughout the 52-week treatment period had a durable response to therapy, while patients who crossed over from placebo to RCI at the end of the double-blind period at week 8 experienced improvements in multiple measures of disease activity during the OLE. The degree of improvement was generally comparable with that observed with RCI treatment during the double-blind phase and was achieved by about 12–16 weeks after RCI was initiated.

The effects of RCI during the 52-week treatment period were evaluated in post hoc analyses using a number of validated outcome measures, including the hSLEDAI, BILAG-2004, CLASI, PGA, SRI, assessments of tender and swollen joints, as well as two novel composite responder indices. Although the primary endpoint of response using the novel index was not met for the double-blind phase,^16^ RCI demonstrated consistent improvement in results across multiple outcome measures, such as the SLEDAI and BILAG over 52 weeks. Consistency of results across multiple measures was seen in the tender and swollen joint count and CLASI scores among patients with counts and scores >0 at baseline. Improvements in the novel organ-specific responder index were similar to those for the revised novel responder index at week 8, but the response rate using the novel organ-specific responder index was considerably lower at week 52 than the rate using the revised index. Inconsistency between the week 52 responder index rates as opposed to the rates at week 8 most likely relates to the longer exposure and thus greater opportunity for mild flares to be captured due to the more stringent BILAG threshold of worsening that was incorporated into the response definition of the novel responder index (no worsening in other organ systems on the basis of BILAG score) versus the revised novel responder index (no new BILAG A score and no more than one new BILAG B organ domain score). BILAG worsening of any degree is a particularly stringent endpoint as minor variations in BILAG disease activity or laboratory variables can too easily trigger worsening domain scores.^24^ The revision defined BILAG worsening based on the original SRI. While stringent analyses are important to demonstrate substantial improvement, they can also be challenging to use to demonstrate treatment effects in small studies. Therefore, this revision to the novel responder index may prove useful in future pilot studies of treatment when looking for indication of a potential response. Taken together, the efficacy results provide preliminary, randomised, controlled evidence that RCI treatment results in reductions in disease activity and corticosteroid requirements in patients with SLE who have persistent disease despite moderate-dose corticosteroid therapy.

RCI was well tolerated in the pilot study. No new safety signals were observed during the 52-week treatment period. Important study limitations include the small sample and the lack of inferential statistical analyses, meaning that conclusions regarding clinical improvement with long-term RCI must be viewed with caution.

In conclusion, the results of this pilot 52-week study support RCI as a treatment option for patients with SLE disease manifestations that are unresponsive to moderate-dose corticosteroid therapy. Preclinical evidence in healthy humans demonstrating direct, steroid-independent effects of RCI on the function of activated human B lymphocytes supports a potential unique mechanism of action for this agent in the treatment of autoimmune diseases characterised by aberrant humoural immune responses.^13^ Additional studies to better understand how differential *in vitro* signaling in response to RCI might contribute to its clinical efficacy are ongoing. Further evaluation of RCI in a well-powered clinical trial in SLE is underway (NCT02953821).
